# A Low-Cost Method for Understanding How Nature-Based Early Learning and Childcare Impacts Children’s Health and Wellbeing

**DOI:** 10.3389/fpsyg.2022.889828

**Published:** 2022-06-23

**Authors:** Oliver Traynor, Anne Martin, Avril Johnstone, Nai Rui Chng, Jessica Kenny, Paul McCrorie

**Affiliations:** MRC/CSO Social and Public Health Sciences Unit, University of Glasgow, Glasgow, United Kingdom

**Keywords:** program theory, play, preschool, nature, outdoors, evaluation, health and wellbeing, children

## Abstract

Nature-based play and learning provision is becoming increasingly popular across the early learning and childcare (ELC) sector in Scotland. However, there remains a lack of understanding of how the program is expected to function. This has implications for program learning and may affect wider rollout of the program. Secondary data analysis of parent interviews (*n* = 22) and observations (*n* = 7) in Scottish ELC settings, and review of internationally published studies (*n* = 33) were triangulated to develop a program theory using the Theory of Change approach. This approach makes a program’s underlying assumptions explicit by systematically demonstrating the relationship between each component: *inputs, activities, outcomes, impact*, and the *contexts* of the program. Findings suggested that location of outdoor nature space, affordances, availability of trained practitioners, and transport to location lead to activities such as free play, educator-led activities, and interactions with nature, resulting in longer durations of physical activity, interactions with peers and educators, and increased engagement with the natural environment. These activities are vital for supporting children’s physical, cognitive, social, and emotional development. Our results demonstrate the value of using secondary data analysis to improve our understanding of the underlying theory of nature-based ELC which can support future evaluation designs. These findings will be of interest to program evaluators, researchers, practitioners, and funders, who find themselves with limited resources and want to better understand their program before investing in an evaluation. We encourage researchers and evaluators in the field of early years and outdoor play in other countries to refine this logic model in their own context-specific setting.

## Introduction

### Early Childhood Nature Experiences

Research suggests that exposure to nature, through outdoor play and learning, can benefit children’s physical and mental development (Tillmann et al., 2018; Truelove et al., 2018; Mygind et al., 2019; Dankiw et al., 2020). Elements of nature such as grassy areas, trees, vegetation, and hilly terrain afford children a variety of play options that can positively impact their physical activity levels, play interaction with their peers and others, emotional resilience, self-esteem, curiosity in nature, and even educational attainment (Becker et al., 2014; Chawla, 2015, 2020; Tremblay et al., 2015; Tillmann et al., 2018; Mygind et al., 2019; Khan et al., 2020). However, most of the evidence speaks to older children and adolescents (i.e., >7-years old); there is less evidence available for younger children (0–7 years), specifically in the preschool/kindergarten setting. Additionally, there is less information regarding the mechanistic pathways outlining how early childhood education programs function and are expected to lead to changes in outcomes (Schindler et al., 2019).

For this paper, and specific to the United Kingdom context, Early learning and childcare (ELC) encompasses all forms of early childhood care for children prior to starting primary school at age 5-years. In the international literature other terms are used such as Early Childhood Education (ECE) or Early Childhood Education and Care (ECEC). Existing evaluations of nature-based ELC settings have a geographical bias with most conducted in the United States, Australia, and Norway (Johnstone et al., 2021). Moreover, they tend to be of poor methodological quality and have a high risk of bias most notably due to small sample sizes, many being uncontrolled interventions and cross-sectional studies, poor recognition of confounding variables and poor reporting of participant dropouts (Dankiw et al., 2020; Johnstone et al., 2021). This affects evaluation results due to not being sufficiently powered to detect a difference, risk of false-positive results, and inability to infer causality.

Moreover, although collaborative steps have been made between researchers and practitioners to better understand the human-nature relationship (Salazar et al., 2021), research in this field still suffers from a lack of understanding of how nature-based play and learning programs in the early years are expected to function and achieve their goals. With no clear conceptualization of the underlying theory of nature-based play and learning programs, it is difficult to assess effectiveness and implementation. A lack of understanding of *how* the program is implemented has implications for the provision of the program across ELC settings and future evaluation designs. Many of these issues could be addressed with a well-developed program theory.

### Program Theory

A program theory is an explicit model of how an intervention or program functions and achieves its goals; firstly through short/intermediate outcomes and then the intended long-term outcomes (Funnell and Rogers, 2011). An explicit program theory details the processes, mechanisms, and circumstances required to achieve change in target outcomes. An evaluation based on program theory will help identify what elements of a program worked and what did not and if other, unaccounted for, aspects (e.g., context-specific factors) influenced how the program contributed to its outcomes (Gervais et al., 2015). Moreover, if a program theory is not present, interpreting the evaluation result may be more difficult since important contextual factors may have been missed and unintended consequences not considered, rendering the results inadequate for future program implementation. By extension, this could limit a program’s ability to inform decision makers (e.g., policy makers and/or urban planners) who need to understand what the active ingredients are.

There are many approaches for developing a program theory such as document reviews, surveys, interviews, workshops, literature reviews and observations (Lam and Skinner, 2021). Although not optimal, re-using data that has been collected for a different primary purpose, in the form of secondary data analysis, is valuable when resources (e.g., time and money) are limited or restrictions on primary data collection are imposed. Additionally, Secondary data analysis demonstrates that program theory development does not require complicated primary data collection methods if a systematic process is followed.

Many funders of evaluation projects, including government and non-government organizations, require a program theory to be submitted for the planning and evaluation of programs (Funnell and Rogers, 2011). However, to our knowledge, program theories are seldom developed using a systematic process with multiple data sources-leading to less rigorous evaluations based on poorly developed program theory. One way of articulating program theory is using a Theory of Change (ToC). The ToC approach makes a program’s *underlying assumptions* explicit by systematically demonstrating the relationship between each component: *inputs, activities, outcomes, impact*, and the *contexts* of the program (Connell and Kubisch, 1998). However, there remains a lack of detailed reporting on the ToC process within the public health literature (Breuer et al., 2016b).

The COVID-19 pandemic has put pressure on educational settings, including the early years sector, to find effective methods to support children’s play and learning while reducing virus transmission and supporting physical distancing. One approach, in the early years, was to increase provision through outdoor settings (Scottish Government, 2021). As well as reducing virus transmission, this approach has promoted more equal access to nature among preschool-aged children and has unveiled the value of outdoor nature-based play and learning. The Scottish Government outlines the different ELC settings that provide nature-based play and learning in its *Out to Play* document (Scottish Government, 2020b). Nature-based play and learning within the Scottish ELC sector is a complex program with multiple pathways likely contributing to child health and wellbeing outcomes. To support the provision of nature-based ELC and ensure future evaluations are viable, it is important to develop a detailed understanding of the program itself. To our knowledge, the literature is missing a well-developed program theory of nature-based ELC in the early years setting.

The aim of this study was to demonstrate the value of developing a program theory of nature-based ELC using secondary data. This paper describes the application of the ToC approach and presents the findings as they relate to: (i) the **resources** required to deliver nature-based play and learning and facilitate time spent outdoors in nature (ii) the **activities** that children take part in while engaging with the program and their associated outputs (iii) the child health and wellbeing **outcomes** associated with attending a nature-based ELC and (iv) the underlying **contextual factors** that influence the provision of time spent outdoors in nature while attending ELC settings in Scotland.

## Materials and Methods

This study used triangulation methodology of three, previously collected, data sources from two independent studies. These two studies were carried out with different aims prior to the initiation of the present study. Secondary analysis is the re-use of data that was collected for a different primary purpose (Heaton, 2008). The decision to conduct secondary data analysis was a pragmatic choice made because of the introduction of national COVID-19 lockdown restrictions in March 2020 at the beginning of this project. This meant that primary data collection with human participants was not possible within the study time frame, therefore secondary data analysis was chosen. Triangulation is the use of more than one data source to address a research question and is often used in mixed-methods studies (O’Cathain et al., 2010). This method encourages researchers to develop a triangulation protocol to display findings and illustrate where findings from each data source agree, partially agree, disagree (dissonance), or where there is silence (findings present in one source but not the other; Farmer et al., 2006; O’Cathain et al., 2010). Silence may occur because of the suitability of a data source to investigate different aspects of a phenomenon (O’Cathain et al., 2010). Using this methodology, we demonstrate how the data addressed each component of the logic model. Three data sources were obtained from two different research projects:

- Interview and focus group transcripts of parents (*n* = 22) whose children attended five different nature-based ELC settings located in the West of Scotland (Project 1—a primary data collection project conducted in 2019).
- Observation schedules of nine outdoor days at *n* = 7 nature-based ELC settings located in the West of Scotland (also from Project 1).
- Published studies extracted from a systematic review (*n* = 33) investigating the relationship between nature-based ELC settings and several child health and wellbeing outcomes across a range of high-income countries (Project 2—a Systematic review project conducted in 2020).

Both Project 1 and Project 2 were conducted independently of the current study. Supplementary Table S1 provides a description of how the secondary data were analyzed for use in the present study.

### Interview and Focus Group Transcripts

Purposeful sampling was used to recruit participants during June/July 2019 to participate in Project 1. Researchers contacted local authority (*n* = 9), partnership (*n* = 4), and private (*n* = 3) ELC settings with outdoor provision in Glasgow (total *n* = 16) and invited them to participate in the study. Of these, 5 agreed to participate (*n* = 2 local authority and *n* = 3 partnership settings) in the interviews and focus groups. Parents (*n* = 17 mothers and *n* = 5 fathers) of children aged 2-4 years attending these five different nature-based ELC settings agreed to take part. During Project 1, parents were told that the purpose of the study was to help researchers understand how parents perceive the role of outdoor ELC for their children’s health and wellbeing and how children spend their time while outside at these settings. Seven individual interviews, three paired interviews, and two focus groups with four parents in each took place. Parents aged from 26 to 48 years and represented a diverse range of socio-economic backgrounds. The interviews and focus groups were conducted by AM and JK, based on an interview guide (see Supplementary Material S6) developed by AM, PM, and JK. The intention of the interviews and focus groups were to explore how nature-based ELC contributes to child and family wellbeing. These were recorded and then transcribed by a professional transcription service who convert focus group and interview recordings into text. For the present study, this data source supported the identification and justification of logic model components including inputs, activities, outcomes, contextual factors, and assumptions.

### Observation Schedules

Of the 16 urban ELC settings referred to above, seven agreed to participate in direct observations. This included the five settings that participated in the interview and focus groups with the addition of two more local authority-managed ELC settings. During June/July 2019, direct observations of 11 nature-based ELC days were carried by one researcher across the seven ELC settings (four settings were observed twice and three were observed once adding up to 11 observations in total) using an observation schedule. The observation schedule was designed by AM, PM, and JK using the Environment Policy and Evaluation Observation (EPAO; Ward et al., 2008) tool as a guide alongside their expert knowledge with the aim to explore how children spend their time at nature-based ELC settings. An example of the observation schedule can be found in Supplementary Material S7. None of the recruited ELC settings had any affiliation with the university. A researcher visited the ELC settings, where possible, on two routine childcare days when they were going to their outdoor location (forest, park, or playground). This was to account for potential variations in observed activities, child-staff interactions, and environmental conditions (e.g., weather). On three occasions however, it was not possible to observe a setting for 2 days due to time constraints. Across all observation days, the minimum length of an observation session was 1 h and 45 min, the maximum was 5 h, and the median was 3 h 50 min. For the present study, analysis of the observation schedules supported the identification and justification of logic model components such as inputs, activities, contextual factors and assumptions. Each round of observations was treated separately within the analysis. Therefore, if the same activity was observed on different days at the same ELC setting, this was considered contextually relevant as a possible component of the logic model. This can be confirmed or refuted during future collaborations with stakeholders.

In total, the observations included 68 children (41 boys and 21 girls) aged 2–5 years. Six of the ELC settings used urban outdoor locations in areas of high deprivation and one ELC setting used an outdoor location with lower level of deprivation.

### Published Studies Extracted From a Systematic Review

The studies were identified from a systematic review on nature-based ELC for child health, wellbeing, and development by co-authors AJ, AM, PM (Johnstone et al., 2021). The review included quantitative and qualitative study designs (e.g., cross-sectional, case–control, randomized, and non-randomized studies) with children (2–7 years) or groups of children as the unit of analysis, nature as the exposure/intervention, traditional ELC settings as the comparison/control and a variety of child health attributes as the outcomes (Johnstone et al., 2021). Full details of the methodology can be found elsewhere (Johnstone et al., 2022a,b).

The studies identified for use in the present paper were selected because they used quantitative or mixed-methods methodology to investigate the impact of nature-based ELC on child outcomes. Therefore, studies that only used qualitative methods were excluded from use in this study. This data source supported the identification and justification of the child outcomes applied to the logic model.

### Analysis

An adapted Framework Method was used to analyze the data (Gale et al., 2013). A coding framework was developed using the logic model categories: inputs; activities; outcomes; and contextual factors. Transcripts were first coded inductively by OT to identify themes which were then grouped into the framework categories demonstrative of the logic model. Transcripts were analyzed using NVivo version 12 qualitative data analysis computer software (QSR International Pty Ltd., 2020).

The observation schedules of each ELC setting were manually analyzed using the coding framework and multiple-colored highlighters by OT. The identified activities, contextual factors, and resources were then added to a framework matrix on Microsoft Excel. This presented the ELC settings as cases (rows) and the observed activities as themes (columns) within the matrix. The framework facilitated analysis of data across and within cases (ELC settings). Activities that were identified three times or more within the observation schedules were taken forward to the triangulation stage. *Outputs* are suggestions by the authors (informed from analysis of the transcripts and observation schedules) as the immediately quantifiable products resulting from taking part in the activities.

The full-text articles of each published study extracted from the systematic literature review were read, and information extracted, including study’s first author, sample size, age group, intervention/exposure, comparator/control, and the outcome(s) of interest. The data from the published studies focused on child health and wellbeing outcomes, such as physical, social, and emotional development, associated with exposure to nature-based ELC. See Supplementary Table S5 for the study characteristics table of the studies analyzed.

### Triangulation Inclusion and Exclusion Criteria

The triangulation protocol is outlined in Table 1. The goal of this program theory development was to design a visual logic model that is broadly representative of the study context while being under constant development. Therefore, the default for triangulating data sources was to include all those that *agreed* or *partially agreed*, unless there were more negative effect directions (i.e., results are not in favor of nature-based ELC within a specific data source) than positive effect directions (i.e., results are in favor of nature-based ELC within a specific data source) associated with a logic model component. For *silence*, if an outcome was discussed in more than half of the interview and focus group transcripts (6 or more), but there was silence from the published studies data, then the outcome was considered contextually specific to the study context and included in the logic model. Any *dissonance* identified was not applied to the logic model.

**Table 1:** Triangulation protocol adapted from Farmer et al., 2006.

Category	Definition*
Agreement	There is almost full agreement between the data sources (e.g., high incidences of logic model component identification and at least 80% of findings from each data source in the positive direction*).
Partial agreement	There is a high incidence of logic model component reporting in one data source but less in the other (e.g., eight incidences in the observation schedules to 1 in the transcript), but both are in the positive direction. Or there is an imbalance of null or negative direction results and positive direction results in the published studies data (e.g., two positive effect studies, one negative effect, and one null effect) alongside positive reporting in the transcripts.
Silence	Only one data source reports on the logic model component (positive direction) and it is not identified in the other data source.
Dissonance	There is disagreement between the data sources. Incidences may be high in both data sources, however, there is a clear difference in effect direction (only negative or null effects in the published studies data compared to positive direction in the transcript data).

* Positive and negative direction: for published studies, a positive direction means findings are in favor of nature-based ELC while negative means not in favor of nature-based ELC and null means no association with nature-based ELC. All of the findings in the observation schedules and interview and focus group transcripts were deemed to be in the positive direction (e.g., children navigating obstacles demonstrates possible positive impact on gross motor development).

Following this protocol, the observation framework matrix was triangulated with the transcript framework matrix in Microsoft Excel. Both matrices were triangulated to identify activities, contextual factors, and resources from each data source that agreed, partially agreed, and disagreed (dissonance) with each other, or if there was information present in one data source but silent in the other. Additionally, the outcomes investigated in the published studies data were listed in an outcomes table and triangulated with the outcomes identified in the transcript analysis. This allowed for the identification of outcomes, from each data source, which agreed with each other, partially agreed, disagreed (dissonance), or where there was silence. The underlying assumptions were extracted from the analysis of the interview and focus group transcripts.

## Results

**Figure 1:**
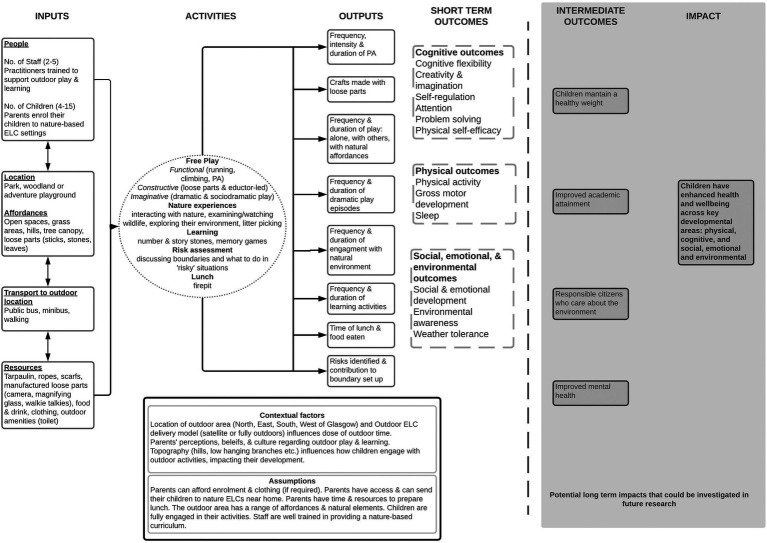
Logic model of a nature-based ELC program in a Scottish urban setting. The figure outlines the inputs required for the activities to take place. The activities produce quantifiable outputs which lead to the measurable short-term outcomes based on the data analyzed in the present study. The outputs are suggestions by the authors based on the evidence demonstrated in this paper. The greyed area show possible intermediate outcomes and long-term impact of the program, however, longitudinal research is required to support these suggestions.

The Theory of Change of a nature-based ELC program is illustrated as a logic model in Figure 1.

### Inputs/Resources

Themes related to inputs that were identified in the analysis of parent interview and focus group transcripts were associated with parents’ organizational skills such as, preparing their child for the day outdoors (e.g., enough warm clothes, lunch, water) and cost of enrolment. A parent also mentioned that children have access to outdoor amenities such as a toilet. Beyond what was reported by parents, the observation data revealed that other resources required to provide outdoor play and learning and support children’s’ activities were natural loose parts (sticks, stones, leaves), manufactured materials (rope swing, cardboard boxes), tarpaulin as cover, and ropes and scarves for setting boundaries.

Analysis of the observation schedules revealed more detail related to the resources and inputs associated with providing outdoor play and learning within Scottish urban ELC settings. These are outlined below:

- The maximum number of children in attendance was 15 with 5 staff members. Across the observations, the average staff to child ratio was 1:3.
- The location of the outdoor setting is an important input (park, woodland, or adventure playground). The observation schedules identified that ELC settings mostly made use of what natural materials were available to them (e.g., fallen trees, natural loose parts, grassy areas, and natural water features) alongside some manufactured materials described above to facilitate children’s play and learning. The observation schedules and transcripts reported on the different risk levels associated with the location. For example, some of the wooded areas used by the satellite settings were popular with dog walkers (signs of dog foul) and some had broken glass.
- Transport to the outdoor location was a crucial resource. ELC settings used a public bus (required walking to bus stop), a minibus, or walked. This is discussed further in the following section.

### Activities

Supplementary Table S2 presents the findings from the triangulation of transcript and observation schedule analysis for the activities section in the logic model development. These data are too extensive to present in this paper, however, to support their understanding and interpretation, an extract from the supplementary table can be found in Table 2. Triangulation of the interview and focus group transcript analysis and observation schedule analysis found agreement across six activities: risk assessment, free play, environmental & nature experiences, educator-led creative activities, learning (literacy and numeracy), and lunch. There was partial agreement with children looking-on/observing. Observation schedules reported this most frequently as young children watching older children play and eventually joining in or copying them. This was considered to be an underlying mechanism of most activities, therefore, was not taken forward as an independent activity in the logic model. The use of fire pits while at nature-based ELC was mentioned once in the transcripts and three times in the observation schedules, therefore, found to only partially agree. Nonetheless, it was only briefly mentioned in one focus group and identified in only three observations where it was used at lunchtime, therefore, the activities “lunch” and “firepit” were merged under “lunch.”

**Table 2:** Extract from triangulation of transcript and observation schedule analysis to determine activities to be included in the logic model.

Activity	Number of transcripts or observation schedules mentioning each activity		Example transcript quote	Example observation	Agree/partial agree/dissonance/silence*	Output
	Transcripts	Observation schedules				
Risk assessment	6	8	*“No mummy. That’s risky business.” You know he really got that very early on and loved teaching me about what was risky and what was safe,”*	*“Discussion of boundaries”*	*Agree*	*Risks identified and contribution to boundary set up*
Free play	9	9	*“they encourage so much free play and then give them chance to kind of explore specific things and the knowledge, as you say, they sort of pick that up”*	*“one child sat in a tree… pretended it was an ice cream van.”* *“Played on a rope bridge over the stream.”*	*Agree*	*Frequency, intensity, and duration of physical activity.* *And play alone and with others.*
Environmental/nature experiences	9	5	“*they were all clustered around examining the frog… he’s got an awareness of them and thinks they are important to be understood and enjoyed like that.”*	“*All were really interested in watching the butterfly”*	*Agree*	*Frequency and duration of engagement with the natural environment*
Travel to outdoor location	0	9	N/A.	“Walk to woods”	*Silence* (only identified in observations)	*N/A (Classified as INPUT)*

* Dissonance suggests disagreement and silence within a data source signifies neither agreement nor disagreement.

N/A, not applicable.

Travel from the ELC setting to the outdoor location by staff and children was identified in all of the observation schedules but there was silence in the transcripts (this was to be expected because this aspect of nature-based ELC is not often considered by parents). It was decided to classify ‘travel to outdoor location’ as an input that is required for nature-based ELC to be delivered optimally. Additionally, this highlights a contextual factor of the nature-based ELC program: sufficiently maintained network of roads and footpaths that facilitate the children and staff to travel from their ELC setting to their outdoor location.

The outputs, as illustrated in Figure 1, were defined by the researchers after data triangulation of the parent interviews and observation schedules confirmed the activities of nature-based ELC. The outputs are the immediately quantifiable products as a result of taking part in the activities. The authors propose recording these outputs to determine progress toward changes in the outcomes illustrated in Figure 1. Any partial agreement or silence between the data sources was not used to define the outputs.

### Outcomes

Table 3 is an extract from the triangulation of focus group and interview transcript analysis triangulated with the published studies data to identify outcomes to be included in the logic model. Supplementary Table S3 has the findings. Across both data sources, three broad themes were identified: (i) cognitive and learning development, (ii) physical development, and (iii) social, emotional, and environmental development.

**Table 3:** Extract from the triangulation of transcript analysis with analysis of the published studies extracted from a systematic literature search to determine outcomes to be included in the logic model.

Outcome	Number of transcripts and published studies highlighting outcome		Example transcript quote	Agree/partial agree/dissonance/silence*
	Transcripts	Published studies		
**Cognitive and learning development**				
Cognitive flexibility	6	1	“I think it makes them more open minded and more creative in their thoughts, because they are able to see things in a different way.”	*Agree*
Attention	5	4	“I mean in great detail, and he has the concentration to do that for that whole two hours aged kind of three and a half…. And with great detail be able to talk about and think and record in his mind what insects are called.”	*Partially agree*
problem solving	7	0	“I do feel that, she is getting more sort of, more abstract learning… You know, it’s more like being resourceful with having nothing.”	*Silence*
**Physical development**				
Physically active	12	14	“He wants to go and like climb up things and just do whatever he’s doing. Run about mental with his brother.”	*Agree*
Gross motor development	5	3	“overall, in the first six months or a year, I saw that her like balance, her like gross motor skills really improved quite a lot.”	*Partially agree*
Illness/ injury	5	4	“(name of child) has got I think quite a good stomach and is not prone to vomiting and diarrhea, she has still got those bugs more in indoor nurseries… but there have been none here [outdoor nursery].”	*Dissonance*
**Social, emotional, and environmental development**				
Social and emotional development	8	7	“(name of child)‘s more able to articulate what she’s feeling and what she sees and what she’s thinking, you know, explain how she’s feeling.	*Agree*
Weather tolerance	10	0	“he does not really bother with the weather, you stick his wellies on and he’s quite happy and I think that’s probably…because he was so outdoorsy at nursery”	*Silence*
Environmental awareness	10	4	“constantly telling me things about insects …He talks to me about pollution…so he’s bringing a lot of stuff back from this [outdoor] nursery which he’s not bringing back from his normal [traditional] nursery”	*Agree*

* Dissonance suggests disagreement and silence within a data source signifies that the outcome was not investigated, therefore, neither agreement nor disagreement.

#### Cognitive and Learning Development

Eight cognitive and learning development outcomes were identified. Of these, there was *agreement* between parent reports in the interview/focus group transcripts and published studies that nature-based ELC benefited cognitive flexibility, creativity and imagination, and self-regulation (Luchs and Fikus, 2013; Müller et al., 2017; Cordiano et al., 2019; Ernst and Burcak, 2019). There was partial agreement between the data sources regarding nature-based ELC being associated with improved children’s attention. The transcript analysis suggested improved attention among children which is in agreement with two published studies (Mårtensson et al., 2009; Ernst and Burcak, 2019) while one study had a negative association and one had null results between nature-based ELC and children’s attention ability (Carrus et al., 2012; Müller et al., 2017). Domains of executive function were investigated in two studies from the published study data (Müller et al., 2017; Ernst and Burcak, 2019). Müller et al. (2017) included attention and working memory under their definition, whilst Ernst and Burcak investigated overall executive function. The analysis of the transcripts revealed no references to executive function’ as an overarching construct (silence). Therefore, this outcome was not applied to the logic model.

Over half of the transcripts discussed improvements in children’s problem solving and physical self-efficacy, however, there was silence in the published studies data. Given the high reporting of these outcomes in the transcripts, they were considered context-specific to the study and applied to the logic model. Finally, applied learning was discussed in three transcripts but not investigated in the published studies data (silence), therefore, this outcome was not applied to the logic model.

#### Physical Development

Four physical development outcomes were identified. An extract of these are shown in Table 3. Of these, there was *agreement* between parent reports in the interview/focus group transcripts and the published studies that nature-based ELC benefited children’s physical activity, and sleep (Boldemann et al., 2006; Storli and Hagen, 2010; Nicaise et al., 2011; Söderström et al., 2013; Cosco et al., 2014; Müller et al., 2017; Torkar and Rejc, 2017; Gubbels et al., 2018; Christian et al., 2019; Määttä et al., 2019; Sando, 2019). Only three studies reported less physical activity (Sugiyama et al., 2012; Olesen et al., 2013; Luchs and Fikus, 2018). *Partial agreement* was identified between the transcripts and published studies that nature-based ELC benefited children’s gross motor development (Fjørtoft, 2004; Müller et al., 2017; Lysklett et al., 2019). This is likely due to the poor quality of current evidence. There is possibly a relationship between physically activity and gross motor competence, however, this requires further investigation.

*Dissonance* was identified between the data sources regarding the impact of nature-based ELC on rates of illness and injury among children. The parent interview and focus group data suggested reduced rates of illness and injury among their children. However, the published studies data found no difference and two studies found a higher incidence of injury within a certain population when comparing nature-based ELC to a traditional ELC setting (Weisshaar et al., 2006; Moen et al., 2007; Söderström et al., 2013; Frenkel et al., 2018). Therefore, this outcome was not applied to the logic model, however, future research should investigate this relationship further.

#### Social, Emotional, and Environmental Development

Three social, emotional, and environmental development outcomes were identified as demonstrated in Table 3. Of these, there was agreement between the interview transcripts and the published studies data that nature-based ELC benefited children’s social and emotional development, and environmental awareness (Carrus et al., 2012; Söderström et al., 2013;Giusti et al., 2014; Park et al., 2016; Müller et al., 2017; Nazaruk and Klim-Klimaszewska, 2017; Sando, 2019). Only two studies found less positive social behavior among children attending nature-based ELC (Cosco et al., 2014; Cordiano et al., 2019).

*Weather tolerance* among children attending nature-based ELC was not investigated in the published study data identified for use in this study, however, it was mentioned across 10 interview and focus group transcripts with parents. Therefore, although triangulation identified *silence* between the two data sources, the outcome *weather tolerance* was still considered important for the context of this study.

### Contextual Factors and Assumptions

#### Contextual Factors

Table 4 demonstrates the triangulation of contextual factors & underlying assumptions from the interview and focus group transcripts and observation schedule analysis. Supplementary Table S4 has additional examples. Three contextual factors were identified. Of these, there was agreement between the interview/focus group data and observation schedule data that the location of the outdoor are and ELC delivery and topography and affordances of the outdoor space are factors that would influence the delivery of nature-based ELC.

**Table 4:** Triangulation of contextual factors and underlying assumptions from analysis transcript and observation schedules.

**Contextual factor**	Number of transcripts or observation schedules mentioning contextual factor or assumption		Example transcript quote	Information from observational data	Agree/partial agree/dissonance/disagree/silence
	Transcripts	Observation schedules			
Location of the outdoor area and ELC delivery model	8	9	“Especially if they go to where they are going and [name] Park is almost at the edge of the city.” “I just live round the corner. So, that was a big factor.”	All ELC settings were based in an urban location. 6 ELC settings were satellite models. 1 was a fully outdoor model.	*Agree*
Parents’ perceptions beliefs, and culture regarding outdoor play and learning	9	0	“something we have encouraged at home as well, is to be, you know, very aware of nature and the need to, you know, kind of protect things and take care of this and, you know, be kind really. That’s the main kind of value we try and instill in our child”	N/A.	*N/A.*
Topography and affordances of outdoor space	4	9	“they used to all get in these, this kind of pallet truck and be dragged along. And it was funny and it was cute at first, but you know, you are really thinking after a while, it’s just quite good for them to kind of like define their own space and investigate it and explore it themselves.”	“all in an open area of the woods with lots of loose parts, leaves, sticks, rocks” “huge tree which had fallen down – children used as a climbing frame”	*Agree*
**Assumptions**					
Parents can afford clothing (if required)	5	0	“The cost of purchasing outdoor wear… wellie boots and the thermal hat, and the thermal socks… that could have been one preventative that could have…stopped me enrolling for an outdoor nursery.”	N/A.	*N/A*
Parents have access to and can send their child to nature ELC settings near home.	6	0	“Like I chose this particular nursery because where I stay.…One) location. Two) it did look like a fun nursery. So, yes, that’s why I chose mine.”	N/A.	*N/A*
Parents have the time and resources to prepare their child’s lunch everyday they are outdoors	4	0	“that [unhealthy food] was a really source of stress for me. It was really important. So, now, although it takes more of my time I provide food for [child] which I think is healthy for her.”	N/A.	*N/A*
Staff are well trained in supporting nature-based play and learning	5	0	“They’re really clear that they want to kind of encourage that strong independent assertive kind of traits in the wee ones…but they look at the positives of kind of non-conformist behaviour”	N/A.	*N/A*

N/A, not applicable.

Finally, *parents’ perceptions, beliefs, and culture regarding outdoor play* and learning was identified in nine transcripts. Due to the nature of the observation schedules, it is not possible to observe parent’s perceptions, nonetheless, this is still considered an important contextual factor to be included in the logic model. These parental factors can have a significant influence over whether their child is enrolled into a nature-based ELC setting. For example, parents in the interviews and focus groups had a variety of social and cultural backgrounds and had their childhoods in different countries (e.g., Russia, Romania, Guatemala, India, and Scotland) which influenced how they thought about the benefits or dangers of playing outdoors.

#### Assumptions

As demonstrated in Table 4, underlying assumptions were only identified in the interview and focus group transcripts, nonetheless, these are considered essential for the nature-based ELC program to function as expected. The underlying assumptions include: parents can afford their child’s outdoor clothing (if required), parents have access to and can send their child to a nature ELC setting near their home, parents have the time and resources to prepare their child’s lunch everyday they are outdoors, and staff are well trained in supporting nature-based play and learning. The final assumption was included because parents often compared practitioner methods across settings (e.g., traditional/indoor vs. nature-based) and mentioned how impressed they were with the resourcefulness of practitioners at nature-based ELC settings and the behaviors they encourage among the children.

## Discussion

To our knowledge, this is the first attempt to define and visually represent the program theory of nature-based ELC. Using triangulation methodology, we have demonstrated how nature-based ELC programs function within a Scottish urban setting, the inputs and resources required to support the activities within this setting, and how the program might exert an effect on children’s health outcomes. Additionally, by outlining how exposure to outdoor play and learning within ELC settings is operationalized, we can acquire a better understanding of the mechanisms that lead to changes in outcomes. This has highlighted the value of a secondary data analysis approach for any researcher wishing to develop a Theory of Change (ToC) of their program. Using these findings, it is possible for ELC practitioners to explore how they might be able to take advantage of their local green space to support children’s play and learning.

The current evidence-base is unable to support ongoing policy decisions in this field, especially related to explicit recommendations such as: dose of nature exposure at ELC settings; minimum and/or optimal environmental affordances required for benefit on child health outcomes; which child health and wellbeing outcomes benefit the most from nature-based play and learning; the activities that support child-led play; and contextual factors that might affect nature-based play and learning implementation (e.g., level of deprivation within the local area). We have attempted to address these issues through our data triangulation process. By using this method, we have demonstrated how to make the ToC explicit. This can now be used as a foundation for researchers and evaluators to identify and test the active ingredients/pathways in the program. Thus, if effectiveness studies support the theorized pathways, the model offers stakeholders the opportunity to make informed funding, policy, and planning decisions. Importantly, these pathways need to be tested formally in an evaluation. Triangulation of the data indicated that nature-based ELC programs could provide children with free play and learning opportunities in nature while supporting development of their cognitive, physical, social, emotional, and environmental outcomes. By engaging in different types of play, interacting with nature, learning activities, and risk assessment through play, children attending urban nature-based ELC may experience improvements in their physical activity levels, gross motor development, and sleep duration. Additionally, children can develop their self-regulation skills, physical self-efficacy, cognitive flexibility, problem solving, attention, creativity and imagination. Finally, children may also experience improved social and emotional development, environmental awareness, and weather tolerance. However, for these experiences to occur there are several underlying assumptions and contextual factors that must be present as demonstrated in the results section and Figure 1.

### Findings in Relation to Other Studies

Although the use of multiple data sources to develop program logic models and Theory of Change (ToC) have been used before, mostly in Evaluability Assessments (Leviton et al., 2010), there is less evidence in the literature regarding the triangulation of data to develop a program theory (Trevisan, 2007; Lam and Skinner, 2021). Where triangulation has been mentioned, such as in the study protocol of a realist evaluation of the Universal Health Visiting Pathway in Scotland, it is not clear whether triangulation of the data sources would be visually represented and how it would inform refinement of the program theory (Doi et al., 2020). Additionally, researchers in Canada applied triangulation methodology in their Evaluability Assessment with a water-based non-governmental organization (Lu et al., 2017). Researchers followed a triangulation protocol defined by Farmer et al. (2006), however it was not clear how many data sources agreed, partially agreed, or disagreed with each other, nor was there any attempt to visually represent the triangulation process (Lu et al., 2017). In this paper, we outlined a formal and transparent process of triangulation that can be used in future studies—improving the replicability of the method.

We identified one study that applied the ToC methodology in a nature-based educational setting (Tiplady and Menter, 2021). Researchers used mixed methods to develop a ToC of how a Forest School program impacted young children’s (primary school aged children) emotional wellbeing. They applied a data triangulation approach to improve the robustness of their results. However, there was little supporting information on the practical implementation of the process. The authors reinforced the benefits of using the ToC approach for identifying important contextual factors that influence change in target outcomes, but there were important differences from the present analysis, specifically the difference in educational setting and age of the study population. The present analysis sheds light on the impact of nature-based ELC on children’s health and wellbeing outcomes, and the contextual factors, inputs, and resources of this institutional setting that influence child development. Furthermore, many of the outcomes we identified are shared by the views of early years professionals interviewed in North America (Beery et al., 2020). For example, interviewed participants from both studies suggested that access to nature in the early years setting can support children’s development of cognitive interest (Beery et al., 2020). Nonetheless, these potential mechanistic pathways need to be tested in an effectiveness evaluation.

### Significance of Our Theory of Change

A theory of change (ToC) is important for further design, implementation, and development of program evaluations. Connell and Kubisch, (1998) propose that a “good” ToC is one that is *plausible, doable*, and *testable. Plausible* refers to the level to which the activities are linked, through existing evidence or inherent logic, to their target outcomes. Mayne (2017) expands upon this, suggesting a ToC must also be robust. To be robust, a ToC must be agreed upon with stakeholders and have assumptions that when recognized can support the program’s implementation. *Do-able* refers to the extent to which the activities are deliverable within the timescale, context, and resources available to the program. Mayne (2017) adds that the effort involved in the activities and outputs should be comparable with the expected results. Finally, *testable* relates to whether the theory is defined enough to support measuring of its progress toward the identified outcomes with acknowledgement of the strength of evidence supporting the results, and assumptions that are unambiguous (Mackenzie and Blamey, 2005; Mayne, 2017). If these criteria can be sufficiently addressed, the ToC can be considered good and robust. These criteria may be considered as guidelines for examining the strength of a ToC and the program it represents while being improved overtime progressing toward a more robust version (Mayne, 2017).

Through the illustrative logic model in Figure 1, we have demonstrated the *plausibility* of our ToC of nature-based ELC. If program implementers apply the inputs and resources illustrated in our logic model, they will be able to provide a variety of nature-based play and learning activities for children to engage with and develop their cognitive, physical, social, emotional, and environmental outcomes. With regards to outcomes, researchers have shown that preschool children who play outdoors in nature spend less time being sedentary and more time being physically active compared with children attending a traditional ELC setting, therefore supporting their physical development (Johnstone et al., 2021). Additionally, active play has been found to be positively and significantly associated with self-regulation in preschool-aged children (Becker et al., 2014). There was also a significant indirect effect between active play and academic achievement through children’s self-regulation (Becker et al., 2014). Therefore, reinforcing the plausibility of our ToC by demonstrating the impact of the program activities on certain outcomes are illustrated in our logic model.

Mayne (2017) stresses the importance of underlying assumptions for the plausibility of a ToC. Our ToC assumes that ELC practitioners are well trained in supporting nature-based play and learning for child development. Researchers have identified how this underlying assumption might be at risk if practitioners have a lack of knowledge regarding how to support preschool children’s play outdoors (McClintic and Petty, 2015). To support the realization of the underlying assumption and the overall plausibility of the ToC, practitioner training around supporting outdoor play and learning in nature is required.

Moreover, our ToC is considered *doable* in the sense that the inputs and contextual information are sufficiently detailed to support the implementation of the program’s activities.

Our findings identified how contextual factors, like topography and location of the outdoor space influence the *do-ability* of program activities. Field observations with twenty-one 3- to 6-year-olds attending a Danish Forest preschool found that forest sites varied with regards to the outdoor features affording children different activities (Lerstrup and Refshauge, 2016). For example, locations with open ground afforded children the opportunity to run around and felled trees afforded climbing. Additionally, distance to the forest site was an important contextual factor and influenced how much time children and practitioners spent at their outdoor location (2–5 h) while availability of practitioners and their professional skills influenced the choice of forest site used on a particular day (Lerstrup and Refshauge, 2016). Similarly, our findings identified potential differences in the characteristics of the outdoor locations. Some of the ELC settings in our study were located in areas of high deprivation. Although not having a direct effect on nature-based ELC provision, area deprivation can have a cumulative effect on the *do-ability* of how an ELC setting functions and supports nature-based play and learning. Research investigating the provision of outdoor play areas across area level deprivation in Glasgow found that more deprived areas had significantly greater number of outdoor play spaces, however, there may be important differences in quality of the outdoor spaces (Ellaway et al., 2007). Therefore, area level deprivation of where an outdoor play area is located could play an important role with regards to children’s exposure to good quality nature and the play affordances available to them. However, this requires further investigation.

Furthermore, research investigating the socio-spatial distribution of walkable environments in Glasgow and Edinburgh found that more deprived areas had greater walkability compared with more affluent areas suggesting that access to nature spaces for children and practitioners may not be any more of a challenge in deprived neighborhoods compared with ELC settings based in less deprived areas (Kenyon and Pearce, 2019). However, the study did not investigate the quality of access (e.g., safety of paths). Further research is required to determine whether the quality of footpath networks connecting ELC settings to their outdoor spaces influences the *do-ability* of implementing the program (e.g., arriving at outdoor space safely). Research in Minnesota found that although most preschools were within 400 m of a greenspace, survey and focus groups identified several contextual barriers associated with access such as ice on the pavements in the winter and misinterpretation of nature play policies (Beery, 2020). This highlights the context-specific nature of do-ability.

The ToC approach has been used for the planning and development of mental health care programs across low- and middle-income countries (Breuer et al., 2016a). The researchers made explicit the influence of context on the do-ability of the program. For example, political buy-in was required to ensure adequate funding and committed leadership for the mental health program to be implemented/doable. The authors accounted for this by having explicit indicators on the pathway to the anticipated outcomes, thus, ensuring that the ToC was testable (Breuer et al., 2016a). In our ToC illustrated in Figure 1, there are explicit outputs, that we have suggested, that can be measured to identify progress toward the outcomes demonstrating the *testability* of the ToC, however, these still require rigorous testing.

Nonetheless, formal stakeholder engagement is required to better assess the do-ability and testability of the ToC. This was not possible in the present study due to the newly implemented COVID-19 national lockdown measures in March 2020. Nonetheless, a ToC should be under constant development and our secondary data analysis approach has been valuable in identifying important contextual information regarding how nature-based ELC is implemented in an urban context.

### Strengths and Limitations

Our methods can help researchers, evaluators, and practitioners in the field of program development and implementation better use finite resources before investing in evaluations. However, generalizability of our findings is not applicable outside of Scotland since two of the data sources (interviews and observation schedules) were specific to the urban Scottish city the study was conducted and the studies extracted from the systematic review were from an international context of high-income countries. Additionally, the original observations were conducted by one researcher, therefore, it was not possible to conduct test reliability rounds to confirm accuracy and consistency of the data recording procedure and the time spent outside varied between ELC settings. This means that there is a risk of bias within the observational data. Moreover, purposeful sampling was used to recruit the ELC settings and the parents who participated during Project 1. It is possible that the ELC settings and parents who chose to participate in the study were not representative of the wider population within the ELC sector. For example, families who value nature less or ELC settings that spend most of their outdoor time in a concrete playground may have chosen not to participate. The interview and focus group sample was also relatively small (17 parents from five ELC settings), approximating 3 parents per ELC setting. Care should therefore be taken if extrapolating these findings more widely.

Moreover, this study did not include the quality score of the published studies used as identified by Johnstone et al. (2021). Future research should investigate the quality of the published studies used as well as the effect direction when determining which outcomes to use in the logic model.

Furthermore, all settings in the observational data required a mode of transport to access the nature space. This may limit the generalizability of the findings to other setting where nature space is available at the premises. Finally, the outputs are only suggestions based on the data we have analyzed and there is not yet enough evidence to confirm the intermediate outcomes and long-term impact illustrated in the logic model. Collaborative work with key stakeholders involved in delivering nature-based ELC is required to further refine the Theory of Change and ensure no key contextual factors or underlying assumptions have been overlooked.

### Implications for Future Research

Importantly, these pathways need to be tested formally in an evaluation.

We have shown how researchers can save costs by using secondary data to develop a program theory rather than spending more money on primary data collection. By having open access to qualitative data within the early years research field, context specific program theories can be developed around the world. We encourage researchers and evaluators in the field of early years and outdoor play to refine this logic model in their own context-specific setting.

Finally, we developed our program theory using secondary data collected pre-COVID-19. Therefore, the Theory of Change will need to be sense-checked with stakeholders from the Scottish ELC context to ensure that it still applies to the present context. This program theory will be continually developed through EA workshops with ELC staff involved in the delivery of the program, identifying key aspects that may have been missed using secondary data analysis alone. These findings will help design a feasibility and pilot study aiming to evaluate nature-based ELC for child health and wellbeing in Glasgow, Scotland. This feasibility and pilot study will address likely key uncertainties such as recruitment methods, randomization methods, and outcome measures, before performing an impact evaluation of the program. This in turn will have implications for policy and practice by informing implementation and rollout of the program across Scotland and improve the available evidence in the academic literature.

## Conclusion

In response to the COVID-19 pandemic, nature-based ELC has been crucial and many ELC settings in Scotland have been looking to further maximize their natural green space (Scottish Government, 2020a). To support implementation of nature-based play and learning, it is important to understand the theory behind the program. This paper has demonstrated the value of developing a program theory using secondary data to improve our understanding regarding the provision of nature-based ELC and its impact on child health and wellbeing. We have demonstrated how urban ELC settings can optimize their local green space to support the development of children’s health and wellbeing with minimal financial investment as long as practitioner numbers are sufficient. We have shown that even when resources and context are limited, a plausible, doable, and testable Theory of Change can and should still be developed. This paper has addressed the issue of poor quality of theory underlying the provision of nature-based ELC. However, stakeholder collaboration is required to refine the program theory, inform future program evaluations, and support the implementation and rollout of nature-based ELC.

## Data Availability Statement

The raw data supporting the conclusions of this article will be made available by the authors, without undue reservation.

## Ethics Statement

The studies involving human participants were reviewed and approved by The College of Medical, Veterinary and Life Sciences Ethics Committee (reference number 200180152), University of Glasgow. Written informed consent to participate in this study was provided by the participants’ legal guardian/next of kin.

## Author Contributions

PM and AM conceptualized and led the qualitative study of interviews and observation schedules. JK contributed to the collection of the qualitative data. AJ, AM, and PM conceptualized and led the systematic review that provided the published studies used in this paper. OT performed analysis of all secondary data sources and wrote each draft of the manuscript. PM, AM, NC, and OT contributed to the conception and theoretical underpinning of the manuscript. All authors contributed to manuscript revision, read, and approved the submitted version.

## Funding

OT was supported by the UK Medical Research Council (funding code MC_ST_00022). AM was supported by the UK Medical Research Council and Scottish Chief Scientific Officer (grant numbers MC_UU_12017/14, MC_UU_00022/1, SPHSU14, SPHSU16). AJ, AM, and PM were supported by the Scottish Government’s Early Learning and Child Care Directorate (grant number 307242–01). PM was supported by the UK Medical Research Council and Scottish Chief Scientific Officer (grant numbers MC_UU_12017/10, MC_UU_00022/4; SPHSU10, SPHSU19). NC was supported by UK Medical Research Council and Scottish Chief Scientific Officer (grant numbers MC_UU_00022/2, SPHSU17). JK was supported by an Wellcome Trust Institutional Strategic Support Fund, University of Glasgow.

## Conflict of Interest

The authors declare that the research was conducted in the absence of any commercial or financial relationships that could be construed as a potential conflict of interest.

## Publisher’s Note

All claims expressed in this article are solely those of the authors and do not necessarily represent those of their affiliated organizations, or those of the publisher, the editors and the reviewers. Any product that may be evaluated in this article, or claim that may be made by its manufacturer, is not guaranteed or endorsed by the publisher.
