# Supplementary material for: A Low-Cost Method for Understanding How Nature-Based Early Learning and Childcare Impacts Children’s Health and Wellbeing
Source: Front Psychol. 2022 Jun 23;13:889828. doi: 10.3389/fpsyg.2022.889828 (PMC9260060; doi:10.3389/fpsyg.2022.889828)
Supplement: Supplementary file 1 [file Table_1.DOCX]

Supplementary Material

Supplementary Table S1: Description of how secondary data sources were used for triangulation

| ***Data source*** | ***Description*** |
| --- | --- |
| Interview & focus group transcripts | 22 parents whose children attended 5 different Scottish nature-based ELC settings took part. Sessions explored how nature-based ELC might contribute to child and family wellbeing and parents’ perception of nature-based play and learning. This data source supported the identification and justification of logic model components including inputs, activities, outcomes, contextual factors, and assumptions. |
| Observation schedules | Direct observations of 7 Scottish nature-based ELC settings across 9 days took place. Details were recorded on the activities children engaged in and their behavior towards peers, staff, and affordances of outdoor location, weather, presence of trees, hills, time spent outdoors in nature, and child to staff ratios. This data source supported the identification and justification of logic model components such as inputs, activities, contextual factors and assumptions. |
| Published studies extracted from a systematic review | 33 studies across several high-income countries were identified from a systematic review on nature-based ELC for child health and wellbeing. The studies used in the present paper implemented quantitative or mixed-methods methodology to investigate the impact of nature-based ELC on child health outcomes. This data source supported the identification and justification of the outcomes applied to the logic model. |

Supplementary Table S2: Triangulation of transcript and observation schedule analysis

| *Activity* | *Number of transcripts or observation schedules mentioning each activity* | | *Example transcript quote* | *Example observation* | *Agree/ Partial agree/ Dissonance/ Silence** | *Output* |
| --- | --- | --- | --- | --- | --- | --- |
|  | *Transcripts* | *Observation schedules* |  |  |  |  |
| Risk assessment | 6 | 8 | *“No mummy. That’s risky business”. You know he really got that very early on and loved teaching me about what was risky and what was safe,”* | *“Discussion of boundaries”* | *Agree* | *Risks identified & contribution to boundary set up* |
| Free play | 9 | 9 | *“they encourage so much free play and then give them chance to kind of explore specific things and the knowledge, as you say, they sort of pick that up”* | *“one child sat in a tree… pretended it was an ice cream van.”*  *“Played on a rope bridge over the stream.”* | *Agree* | *Frequency, intensity, &duration of physical activity.*  *And play alone and with others.* |
| Environmental/nature experiences | 9 | 5 | “*they were all clustered around examining the frog… he’s got an awareness of them and thinks they’re important to be understood and enjoyed like that.”* | “*All were really interested in watching the butterfly”* | *Agree* | *Frequency & duration of engagement with the natural environment* |
| Educator-led creative activities | 5 | 4 | *“… they were hammering… they made sort of dyed material, just like cloth bits, and they made sort of handmade flowers and leaves to sort of produce the imprint…”* | *“Collected leaves/flowers/moss and stuck them to paper to make collages”* | *Agree* | *Crafts made with loose parts* |
| Learning (literacy & numeracy) | 5 | 5 | *“Even like this numbers and letters. It’s all incorporated into the kind of outdoorsy routine… So, counting stones or whatnot. Like, finding letters written on them…”* | “Counting trees & estimating height… playing with story stones.” | *Agree* | *Frequency & duration of learning activities* |
| Travel to outdoor location | 0 | 9 | N/A. | “Walk to woods” | *Silence* (only identified in observations) | *N/A (Classified as INPUT)* |
| Lunch | 3 | 8 | “It is really simple lunches and the thermos [provided by parents] has like either baked beans or soup” | “Lunch… sandwiches provided by staff” | *Agree* | *Time of lunch and food eaten* |
| Yoga | 2 | 0 | “it makes everyone a bit more calm, they do yoga and everything.” | N/A. | *Silence* (only identified in transcripts) | *N/A* |
| Looking-on/ observing | 1 | 8 | “he’s following 3 to 5s about, just watching what they were doing.” | “a group of girls were doing this [climbing & jumping] but the boys joined in once they noticed them.” | *Partial agreement* (mostly present in observation schedules) | *N/A. Considered an underlying mechanism* |
| Fire pit (cooking food) | 1 | 3 | “Yeah I think they had that [fire]… marshmallows.” | “cooked…popcorn, & hot dogs over fire.” | *Partial agreement* | *N/A. Categorized under lunch* |
| *Dissonance suggests disagreement and silence within a data source signifies neither agreement nor disagreement. N/A = not applicable. | | | | | | |

Supplementary Table S3: Triangulation of FG & interview transcript analysis & published studies data analysis

| *Outcome* | *Number of transcripts & Empirical studies exploring/investigating outcome* | | *Example transcript quote* | *Agree/ Partial agree/ Dissonance/ Silence** |
| --- | --- | --- | --- | --- |
|  | *Transcripts* | *Published studies* |  |  |
| ***Cognitive & learning development*** | | | | |
| Cognitive flexibility | 6 | 1 | “I think it makes them more open minded and more creative in their thoughts, because they’re able to see things in a different way.” | *Agree* |
| Creativity & imagination | 6 | 3 | “she started to make up stories. So, her imagination is really, is really over the top. Yeah, that’s something that happened in the outdoor nursery.” | *Agree* |
| Self-regulation | 5 | 2 | “thought today coming…because he’s been saying to me that, “(name of friend) is not coming anymore….So that is going to make it harder for him. But he was fine this morning. He needed about seven minutes of reassurance and then he just got up and started to play…” | *Agree* |
| Attention | 5 | 4 | “I mean in great detail, and he has the concentration to do that for that whole two hours aged kind of three and a half…. And with great detail be able to talk about and think and record in his mind what insects are called.” | *Partially agree* |
| problem solving | 7 | 0 | “I do feel that, she is getting more sort of, more abstract learning… You know, it’s more like being resourceful with having nothing.” | *Silence* |
| Executive function | 0 | 2 | *N/A.* | *Silence* |
| Applied learning | 3 | 0 | “No, mummy. That’s risky business. You know he really got that very early on and loved teaching me about what was risky and what was safe.” | *Silence* |
| Physical self-efficacy | 7 | 0 | “[child is] confident in his own ability to know when to, he’s got too high on something or he can’t do something. He won’t push … he’ll know but he’s sort of success rate is pretty good, but he’ll know when something is going to be beyond him, which I think is a good thing” | *Silence* |
| ***Physical development*** | | | | |
| Physically active | 12 | 14 | “He wants to go and like climb up things and just do whatever he’s doing. Run about mental with his brother.” | *Agree* |
| Gross motor development | 5 | 3 | “overall, in the first six months or a year, I saw that her like balance, her like gross motor skills really improved quite a lot.” | *Partially agree* |
| Sleeping | 10 | 1 | “She was more, definitely more tired, on those days. She did manage to go to her bed a bit earlier. Sometimes it’s a struggle to get her to stay up until bed time…” | *Agree* |
| Illness/ injury | 5 | 4 | “I would say, given that (name of child) has got I think quite a good stomach and isn’t prone to vomiting and diarrhea, she has still got those bugs more in indoor nurseries… but there have been none here [outdoor nursery].” | *Dissonance* |
| ***Social, emotional, & environmental development*** | | | | |
| Social & emotional development | 8 | 7 | “(name of child)’s more able to articulate what she’s feeling and what she sees and what she’s thinking, you know, explain how she’s feeling. Rather than just making noises.” | *Agree* |
| Weather tolerance | 10 | 0 | “he doesn’t really bother with the weather, you stick his wellies on and he’s quite happy and I think that’s probably a lot to do with it to be honest because he was so outdoorsy at nursery” | *Silence* |
| Environmental awareness | 10 | 4 | “constantly telling me things about insects …He talks to me about pollution…so he’s bringing a lot of stuff back from this [outdoor] nursery which he’s not bringing back from his normal [traditional] nursery. So I think he’s getting a lot of learning …” | *Agree* |
| *Dissonance suggests disagreement and silence within a data source signifies that the outcome was not investigated, therefore, neither agreement nor disagreement | | | | |

Supplementary Table S4 Triangulation of contextual factors & underlying assumptions

| *Contextual factor* | *Number of transcripts or observation schedules mentioning contextual factor or assumption* | | *Example transcript quote* | *Information from observational data* | *Agree/ Partial agree/ Dissonance/ Disagree/ Silence* |
| --- | --- | --- | --- | --- | --- |
|  | *Transcripts* | *Observation schedules* |  |  |  |
| Location of the outdoor area and ELC delivery model | 8 | 9 | “Especially if they go to where they’re going and [name] Park is almost at the edge of the city.”  “I just live round the corner. So, that was a big factor.” | All ELC settings were based in an urban location. 6 ELC settings were satellite models. 1 was a fully outdoor model. | *Agree* |
| Parents’ perceptions beliefs, & culture regarding outdoor play & learning | 9 | 0 | “before I moved back to Glasgow, I was living in Guatemala and living in a really rural community. Where the kids just ran free from aged two basically. And the levels of happiness and communality and friendship and just wellbeing of those kids, was so much greater than what I see in Glasgow.”  “something we have encouraged at home as well, is to be, you know, very aware of nature and the need to, you know, kind of protect things and take care of this and, you know, be kind really. That's the main kind of value we try and instill in our child” | N/A. | *N/A.* |
| Topography & affordances of outdoor space | 4 | 9 | “they used to all get in these, this kind of pallet truck and be dragged along. And it was funny and it was cute at first, but you know, you’re really thinking after a while, it’s just quite good for them to kind of like define their own space and investigate it and explore it themselves.” | “all in an open area of the woods with lots of loose parts, leaves, sticks, rocks”  “huge tree which had fallen down – children used as a climbing frame” | *Agree* |
| *Assumptions* | *Number of transcripts or observation schedules mentioning contextual factor or assumption* | | *Example transcript quote* | *Information from observational data* | *Agree/ Partial agree/ Dissonance/ Disagree/ Silence* |
|  | *Transcripts* | *Observation schedules* |  |  |  |
| Parents can afford clothing (if required) | 5 | 0 | “The cost of purchasing outdoor wear… wellie boots and the thermal hat, and the thermal socks… that could have been one preventative that could have…stopped me enrolling for an outdoor nursery.” | N/A. | *N/A* |
| Parents have access to and can send their child to nature ELC settings near home. | 6 | 0 | “Like I chose this particular nursery because where I stay. …One) location. Two) it did look like a fun nursery. So, yes, that’s why I chose mine.” | N/A. | *N/A* |
| Parents have the time and resources to prepare their child’s lunch everyday they’re outdoors | 4 | 0 | “that [unhealthy food] was a really source of stress for me. It was really important. So, now, although it takes more of my time I provide food for [child] which I think is healthy for her.” | N/A. | *N/A* |
| Staff are well trained in supporting nature-based play & learning | 5 | 0 | “They’re really clear that they want to kind of encourage that strong independent assertive kind of traits in the wee ones…but they look at the positives of kind of non-conformist behaviour” | N/A. | *N/A* |
| N/A = not applicable. | | | | | |

Supplementary Table S5: Study characteristics of the published studies extracted from a systematic review study used in the present paper

| **Reference number** | **Author & year** | **Participant age group (years)** | **Country** | **Study duration** | **Summary (sample size, number of settings, exposure (E), comparison (C), outcomes)** |
| --- | --- | --- | --- | --- | --- |
|  | ***Controlled pre- & post-test studies*** | | | | |
| [1] | Cordiano et al., 2019 | 4 | USA | 8 months | E: 12 children in nature-based ELC program. C: 14 children in traditional ELC program. All children were girls and from the same school.  Researchers quantitively compared the groups regarding social interaction, play, behavior, school enjoyment, and nature relatedness. |
| [2] | Ernst & Burcak 2019, USA | 4 | USA | 9 months | E: 84 children across 4 ELC settings. C: 24 children across 2 ELC settings. 50% girls/ 50% boys. Researchers investigated curiosity outcomes.  All nature ELC settings, across the four studies, used a combination of wild natural settings that were minimally managed and natural playscapes designed for nature play. Most of the time was spent in unmaintained or minimally maintained natural settings regardless of weather conditions (roughly 4-5 hours/day)  Both non-nature ELC settings emphasised child-directed play with most of the time spent indoors in free or loosely guided play (4-5 hours). Children at both settings had 1-2 hours of daily outdoor playtime (weather permitting) in a maintained outdoor space with playground equipment. |
|  | Zamzow & Ernst, 2020  Controlled before & after | 4 | USA | 9 months | E: 78 children across 4 ELC settings. C: 44 children across 2 ELC settings.  Description as above.  Researchers investigated cognitive outcomes e.g., executive functioning skills. |
|  | Wojciehowski & Ernst (2018) uncontrolled before & after | 4 | USA | 9 months | E: 75 children across 4 ELC settings.  Description as above.  Researchers investigated creativity outcomes. |
|  | Ernst et al 2019, uncontrolled before & after | 4 | USA | 9 months | E: 78 children across 4 ELC settings.  Description as above.  Researchers investigated resilience outcomes. |
| [3] | Fjørtoft 2004, Norway | 5-7 | Norway | 10 months | E: 46 children in a single ELC setting. C: 29 children in 2 ELC settings. 37 girls and 38 boys.  E: Children used the forest next to the kindergarten every day for up to 2 hours. Sometimes, the outdoor playground inside the preschool fence was used. The forest included mixed woodland vegetation, open spaces and meadows.  C: Children used the traditional outdoor playground for up to 2 hours per day and sometimes visited natural sites.  Researchers investigated children’s motor skills. |
| [4] | Müller et la., 2017, Canada, | 4-5 | Canada | 9 months | E: 43 children at one nature kindergarten. C: 45 children at one traditional kindergarten. Settings and study population are not described.  Researchers investigated children’s physical activity, motor skills, cognitive outcomes, social & emotional outcomes, nature relatedness. |
|  | ***Uncontrolled pre- & post-test studies*** | | | | |
| [5] | Cosco et al., 2014 | 2-5 | USA | Not reported. | E: 804 children across 27 ELC settings.  Preventing Obesity by Design is an ELC outdoor redevelopment program. Pre-redevelopment, the outdoor space had minimal structures (slides, swings) contained within an enclosed rectangular space. Post-redevelopment, the space contained more natural elements, including trees and vegetation.  Researchers investigated physical activity, social & emotional outcomes. |
| [6] | Nazaruk & Klim-Klimaszewska, 2017 | 6 | Poland | 6 months | E: 90 children. 50 children with their ELC setting in an urban setting. 50 children with the ELC setting in a rural setting.  Teachers organised trips to the forest, park, allotment garden, meadows, agritourism farm, and zoos.  Researchers investigated nature relatedness. |
| [7] | Park et al., 2016 | 5-7 | South Korea | 24 weeks | E: 336 children across 12 settings. 167 girls, 169 boys.  Intervention was composed of horticultural activities with the aim of improving children’s knowledge of the process between seeds and planting to harvesting. 24 sessions were delivered once per week and lasted around 50 minutes.  Researchers investigated cognitive, social, & emotional outcomes, and science investigation abilities and attitudes. |
|  | ***Controlled cross-sectional studies*** | | | | |
| [8] | Cloward Drown et al., 2014 | 3-5 | USA | N/A. | E: 24 children in observed in 1 ELC across 2 different playgrounds (natural vs manufactured). 17 girls and 7 boys.  E: natural playground consisted mostly of natural surfaces (vegetation, boulders, grass) but also had sandboxes, bike paths, and manufactured loose parts.  C: manufactured playground contained a variety of equipment with hard surfaces. Some vegetation was present, main features were a xylophone, slide and pit, water play area, ball pit, concrete ramps leading to a plastic play castle and spin chair.  Researchers investigated play outcomes. |
| [9] | Giusti et al., 2014 | 5 | Sweden | N/A. | E: 11 children across 2 ELC settings. C: 16 children across 5 ELC setting.  ELC settings were assessed on their affordances and accessibility of nature experiences. ELC settings with contrasting accessibility were selected.  E: The 10 ELC settings with the greatest accessibility and most frequent use of nature experiences.  C: The 10 ELC settings with the lowest accessibility and least frequent use of nature experiences.  Researchers investigated children’s nature relatedness. |
| [10] | Lysklett et al., 2019 | 5-6 | Norway | N/A. | E: 43 children across 4 ELC settings. C: 49 children across 4 ELC settings. 39 girls and 53 boys.  Nature-based ELC settings located close to a large recreational area with woods, lakes and tracks just outside the city centre. Both types of preschools used the nearby area for hiking and play every week,  E: nature-based ELC settings used the recreational area at least 3 times per week.  C: traditional ELC settings used the recreational area once per week.  Researchers investigated motor competence and physical fitness. |
| [11] | Luchs & Fikus, 2013 | 5-6 | Germany | N/A. | E: 38 children at one ELC setting. C: 21 children at one ELC setting. 26 girls and 33 boys.  E: the nature-based playground afforded children wild and natural areas including tress, grass, and vegetation. Also available were sandboxes, dirt, rock, water, and mud area.  C: the traditional playground was mostly manufactured structures including slides, sandbox, playhouse, seesaw, roundabout etc.  Researchers investigated children’s play outcomes. |
| [12] | Robertson et al., 2020 | 4-5 | Australia | N/A. | E: 15 children at one ELC setting. C: 15 children at one ELC setting.  E: Rural ELC setting will a small traditional playground (sand pit, obstacle courses etc.) and a large open ended natural area (trees, shrubbery, grass, natural loose parts).  C: Suburban ELC setting with mostly manufactured structures with some natural elements such as trees and vegetable garden.  Researchers investigated children’s play outcomes. |
| [13] | Rice & Torquati, 2013 | 2-5 | USA | N/A. | E: 68 children across 6 ELC programs. C: 46 children across 4 ELC programs.  E: Nature programs contained vegetation, gardens, areas for digging soil, sand, and loose parts (sticks, rocks, wood, seeds, pine cones) and other naturally occurring objects that children used in play. There was also built climbing structures such as a boat and playhouse.  C: Non-nature programs were made-up of mostly pretend play structures, sand and/or wood chips, paved surfaces for wheeled toys, and some natural elements including trees or grass.  Researchers investigated children’s nature relatedness. |
| [14] | Moen et al., 2007 | 3-6 | Norway | N/A. | E: 267 children across 37 ELC settings. C: 264 children across 32 ELC settings.  E: ELC settings had “outdoor” or “nature” as part of their name or stressed outdoor pedagogy. Children spent an average of 3.5-8 hours/day outdoors in winter.  C: ELC settings where children spent an average of 1.25-4 hours/day outdoors.  Researchers investigated rates of illness. |
| [15] | Weisshaar et al., 2006 | 4 | Germany | N/A. | E: 506 children across 25 ELC settings. C: 1201 children across 28 ELC settings. 803 girls and 901 boys.  E: nature-based ELC setting located in a forest. Children spend all season outdoors.  C: traditional ELC settings (not described).  Researchers investigated rates of illness. |
| [16] | Frenkel et al., 2019 | 2-5 | USA | N/A. | E: 71 children across 5 ELC settings. C: 70 children across 4 ELC settings. 59 girls and 82 boys.  E: Nature-based ELC settings were situated in parks containing marked off areas with rocks and natural features for daily activities (grassy areas, dirt, tree cover, sticks etc.)  C: Traditional ELC setting had outdoor play areas built on concrete. Children spent less than 1.5 hours outdoors each day.  Researchers investigated rates of illness. |
|  | ***Cross-sectional studies*** | | | | |
| [17] | Boldeman et al., 2004 | 1-6 | Sweden | N/A. | E: 64 children across 2 ELC settings. 38 girls and 26 boys.  E: The first ELC setting had play scaffolding surrounded by trees but expose to the sun  The second ELC setting had play scaffolding under a tree canopy.  Researchers investigated UV exposure. |
| [18] | Carrus, 2012 | 1-3 | Italy | N/A. | E: 16 children at 1 ELC setting.  Free play in the ELC setting’s garden and green spaces compared to free play indoors.  Researchers investigated children’s cognitive, social, and emotional outcomes. |
| [19] | Storli etl al., 2010 | 3-5 | Norway | N/A. | E: 16 children at 1 ELC setting. 7 girls and 9 boys.  Observations took place while children played in their traditional playground (composed of slides, swings, grassy hills, sandpit, swings) compared with when children played in a natural environment (on a pebbled beach and rugged slopes – children gathered loosed parts, climbed and ran about).  Researchers investigated children’s physical activity. |
| [20] | Morrissey et al., 2017 | 4-5 | Australia | N/A. | E: 28 children at one ELC setting. C: 28 children from the same ELC setting as E. 28 girls and 28 boys.  E: ELC play space was highly naturalised with large trees, open grassy areas, dry creek bed, logs, steppingstones, and a few manmade structures.  C: A traditional play space with fixed structures such as ladders, swings, tunnels, concrete path, tyre swings, and climbing frames and some natural elements such as trees.  Researchers investigated children’s play outcomes. |
| [21] | Luchs & Fikus 2018, Germany | 5-6 | Germany | N/A. | E: 17 children at 1 ELC setting. 8 girls and 9 boys.  Children were observed in differently designed playgrounds.  E: Natural playground was a large space with trees, grass, hills, vegetation and water.  C: Traditional playground had manmade structures such as slides and swings with some natural elements (grass).  Researchers investigated children’s physical activity. |
| [22] | Torkar & Rejc, 2017 | 4-5 | Slovenia | N/A. | E: 25 children at 1 ELC setting.  Children were observed on a traditional playground (C) and a forest playground (E).  E: Forest playground contained was composed of trees, river bushes, trails, and fallen tree trunks.  C: Traditional playground contained manufactured features such as a playhouse, slide, sandbox, seesaw, and roundabout.  Researchers investigated children’s physical activity. |
| [23] | Boldemann et al., 2006 | 4-6 | Sweden | N/A. | E: 199 children across 11 ELC settings. 85 girls and 114 boys.  Outdoor environments were assessed on their availability of play opportunities based size of space, overgrown surfaces (trees & shrubbery) and integration of play structures.  Researchers investigated children’s physical activity and UV exposure. |
| [24] | Christian et al., 2019 | 3-5 | Australia | N/A. | E: 678 children across 48 ELC settings. 47% girls.  ELC settings were assessed based on the location, shape, and size of outdoor play space and then categorised.  Researchers investigated children’s physical activity and UV exposure. |
| [25] | Dyment 2013, Australia | 2-5 | Australia | N/A. | E: 120 children across 3 ELC settings. C: 40 children at 1 ELC setting. 43% girls.  E: The 3 ELC settings contained natural play spaces (trees, rocks, gardens). Two also had manufactured features.  C: One ELC setting which contained no natural play spaces.  Researchers investigated children’s play outcomes. |
| [26] | Gubbels et al., 2018 | 1-3 | Netherlands | N/A. | E: 151 children across 22 ELC settings. 9 girls and 72 boys.  The childcare environments were assessed using a standardised observation protocol. Associations between the childcare environment and children’s sedentary behavior and physical activity we examined. |
| [27] | Mååttå et al., 2019 | 3-6 | Finland | N/A. | E: 864 children across 66 ELC settings. 48% girls.  The childcare environments were assessed based on environmental affordances.  Researchers investigated children’s physical activity. |
| [28] | Mårtensson 2009, Sweden | 4-6 | Sweden | N/A. | E: 198 children across 11 ELC settings. 85 girls and 113 boys.  The outdoor settings of each ELC setting were assessed based on their natural affordances and then categorised into low or high scoring groups.  Researchers investigated children’s cognitive outcomes. |
| [29] | Nicaise et al., 2011 | 4-5 | USA | N/A. | E: 51 children from one ELC setting. 28 girls  Children were observed, using a standardised protocol, in two outdoor play areas containing natural features (grass and trees) and manufactured features (slide, sandbox, seesaw, climbing frames etc.)  Researcher’s investigated children’s physical activity. |
| [30] | Olesen et al., 2013 | 4-6 | Denmark | N/A. | E: 441 children across 42 ELC settings. 51% girls 49% boys.  Researchers collected environmental correlates such as vegetation and hilly landscape and tested for correlation with children’s physical activity levels. |
| [31] | Sando 2019 | 2-4 | Norway | N/A. | E: 80 children across 8 ELC settings. 39 girls and 41 boys.  The outdoor environment of ELC settings we categorised to sandbox, pathways, nature, open area, fixed equipment, and indoor.  Nature ranged from large forest areas to small areas with trees and natural surfaces.  Researchers investigated children’s physical activity, social and emotional outcomes. |
| [32] | Söderström et al., 2013 | 3-5 | Sweden | N/A. | E: 172 children across 9 ELC settings.  Outdoor environment of ELC settings were assessed using a standardised outdoor play environment assessment tool and categorised based on the total availability of outdoor area, number of trees, shrubbery, and hilly terrain, integration between open areas, vegetation, and play structures.  Researchers investigated children’s sleep, injury, weight status, and social & emotional outcomes. |
| [33] | Sugiyama et la., 2012 | 3-5 | Australia | N/A. | E: 89 children across 10 ELC settings. 46% girls and 54% boys.  ELC setting directors completed questionnaires assessing the settings characteristics such as vegetation, gradient of shade, and surface materials (grass).  Researcher’s investigated children’s physical activity. |

Supplementary material S6

Focus Group & Individual Interview guide

**Study title: Exploring the potential of outdoor nurseries for health and wellbeing of children and families**

Topic 1: Factors behind choice of enrolling children in outdoor nursery

- What motivated you to enrol your child/ren in the outdoor nursery?
- Which factors influenced your choice in enrolling your child in an outdoor nursery? (probe: closeness to home, belief of benefits, recommendation by other people)
- Do any of your friends’ or neighbours’ children attend outdoor nurseries? If so, to what extent do you think did this influence your decision to sign up for outdoor nursery child care?
- Were there any barriers related to enrolment that you had to overcome? (probe: long waiting list, distance to home, objection by family members and/or the child)
- What could have prevented you from enrolling your child in the outdoor nursery? (Probe: perception of risks for injuries, weather, sickness)

Topic 2: Perception and understanding of benefits of outdoor nurseries for health and wellbeing

- To your understanding, how can outdoor nurseries be good for your child? (Probe: learning, physical health, sleep, self-esteem, problem-solving skills, social interactions with others, etc.)
- What kind of benefits have you observed for your child’s health and wellbeing? (Probe: as above)
- Are there any benefits that go beyond benefits for your child, for example for you and/or other family members? (Probe: parenting, family relationships, physical activity levels - siblings, other parent, grandparents)
- Compared to before your child/children were attending the outdoor nursery, to what extent has your time spend outdoors/in nature/green space changed?
- To what extent has your social network, that is the people you spend time with/talk to, changed since your child/ren attend the outdoor nursery? (Probe: increased in size - more friends; spending time with families whose children are in outdoor nurseries as well, people who are connected to nature, are generally physically active, etc)

Topic 3: Unintended negative consequences of outdoor nursery child care

- Can you think of any negative consequences or events associated with attending outdoor nurseries for your child’s health and wellbeing?

Topic 4: Post enrolment beliefs, issues, and concerns (if not already arisen as part of other topics)

- Having experience with your child(ren) being in an outdoor nursery, does it live up to what you expected?
- Can you think of anything you would change with regard to the outdoor nursery provision your child receives?
- Anything else that explores whether parents still hold similar motivations as they did prior to enrolment, nursery capacity, service provision etc

| *Supplementary material S7: Example observation schedule* | | | | | | | | | | | | | | |
| --- | --- | --- | --- | --- | --- | --- | --- | --- | --- | --- | --- | --- | --- | --- |
| **Nursery ID:** | **Observation day:** | | **Date:** | | **Weekday:** | | **Number of teachers:** | | **Number of children (girls:boys):** | | | | **Age group (years):** | |
|  | | **Time of day** | | | | | | | | | | | | |
| **Items to be observed** | | **Morning** | | | | | | **Afternoon** | | | | | | |
| **Time and duration of activity (minutes)** | |  | |  |  |  | |  | |  |  |  | |  |
| **Description of activity** | | *E.g., climbing up tree* | |  |  |  | |  | |  |  |  | |  |
| **Purpose of activity** | | *E.g., Physical activity/ risky play* | |  |  |  | |  | |  |  |  | |  |
| **Staff-instructed activity** | | *E.g., No* | |  |  |  | |  | |  |  |  | |  |
| **Child-directed activity** | | *E.g., Yes* | |  |  |  | |  | |  |  |  | |  |
| **Description of outdoor area/ how it is used** | | *E.g., A tree that had many branches which afforded climbing* | |  |  |  | |  | |  |  |  | |  |
| **Teamwork/communication between children** | | *E.g., One child started climbing while others observed and then joined* | |  |  |  | |  | |  |  |  | |  |
| **Interaction between staff and children** | | *E.g., Staff cautiously observed* | |  |  |  | |  | |  |  |  | |  |
| **Interest in/comment about environment** | | *E.g., child commenting on the size of the tree* | |  |  |  | |  | |  |  |  | |  |
| **Children’s attitude towards activity/ behaviour** | | *E.g., Appear happy and confident in ability to climb tree* | |  |  |  | |  | |  |  |  | |  |
| **Child/ren reflect on own ability** | | *E.g., Reflecting on how high they climbed* | |  |  |  | |  | |  |  |  | |  |
| **Differences between children (age/gender)** | | *E.g., only girls were observed climbing* | |  |  |  | |  | |  |  |  | |  |
| **Description of weather** | | *E.g., Sunny* | |  |  |  | |  | |  |  |  | |  |
